# Efficient large-scale generation of functional hepatocytes from mouse embryonic stem cells grown in a rotating bioreactor with exogenous growth factors and hormones

**DOI:** 10.1186/scrt356

**Published:** 2013-12-02

**Authors:** Shichang Zhang, Yunping Zhang, Li Chen, Tao Liu, Yangxin Li, Yingjie Wang, Yongjian Geng

**Affiliations:** 1 Artificial Liver Lab., Southwest Hospital, Third Military Medical University, Chongqing 400038, China; 2 University of Texas Health Science Center and Texas Heart Institute, Houston, TX 77030, USA; 3 Department of Emergency Medicine, Shanghai No. 6 Hospital, JaoTong University, Shanghai 200024, China; 4 Department of Obstetrics and Gynecology, The First Affiliated Hospital of Nanjing Medical University, Nanjing, Jiangsu 210017, China; 5 Department of Internal Medicine 3, The Northern Region of No.401 Hospital, Qingdao, Shandong 266100, China

## Abstract

**Introduction:**

Embryonic stem (ES) cells are considered a potentially advantageous source of hepatocytes for both transplantation and the development of bioartificial livers. However, the efficient large-scale generation of functional hepatocytes from ES cells remains a major challenge, especially for those methods compatible with clinical applications.

**Methods:**

In this study, we investigated whether a large number of functional hepatocytes can be differentiated from mouse ES (mES) cells using a simulated microgravity bioreactor. mES cells were cultured in a rotating bioreactor in the presence of exogenous growth factors and hormones to form embryoid bodies (EBs), which then differentiated into hepatocytes.

**Results:**

During the rotating culture, most of the EB-derived cells gradually showed the histologic characteristics of normal hepatocytes. More specifically, the expression of hepatic genes and proteins was detected at a higher level in the differentiated cells from the bioreactor culture than in cells from a static culture. On further growing, the EBs on tissue-culture plates, most of the EB-derived cells were found to display the morphologic features of hepatocytes, as well as albumin synthesis. In addition, the EB-derived cells grown in the rotating bioreactor exhibited higher levels of liver-specific functions, such as glycogen storage, cytochrome P450 activity, low-density lipoprotein, and indocyanine green uptake, than did differentiated cells grown in static culture. When the EB-derived cells from day-14 EBs and the cells’ culture supernatant were injected into nude mice, the transplanted cells were engrafted into the recipient livers.

**Conclusions:**

Large quantities of high-quality hepatocytes can be generated from mES cells in a rotating bioreactor via EB formation. This system may be useful in the large-scale generation of hepatocytes for both cell transplantation and the development of bioartificial livers.

## Introduction

The management of patients with acute liver failure (ALF) is challenging. Emergency liver transplantation remains the most successful treatment in many cases of ALF. However, because of the shortage of available donor organs, only 20% of patients with ALF receive a transplant, and 80% die while on the waiting list [1]. In the past decade, both hepatocyte transplantation and bioartificial livers have been investigated as a “bridge” or alternative to liver transplantation, which are promising treatment options for ALF patients awaiting a donor liver [2-4]. However, hepatocyte transplantation and the development of bioartificial livers entail a large quantity of high-quality hepatocytes, which also requires a donor liver. Thus an urgent need exists for an alternative and adequate supply of suitable hepatocytes for both hepatocyte transplantation and bioartificial livers [5].

Embryonic stem (ES) cells, pluripotent cells derived from the inner cell mass of preimplantation blastocysts, have the unique ability to give rise to all somatic cell lineages [6,7]. In particular, the differentiation of hepatocytes or hepatocyte-like cells from ES cells *in vitro* and *in vivo* has been reported [8-10]. Such ES cell-derived hepatocytes are anticipated to be a useful source of cells for the treatment of liver diseases [11,12]. However, none of the previous work with ES cells has achieved the efficient differentiation of ES cells into hepatocyte-like cells by using a protocol that meets the demands of the clinical applications.

Recent studies have demonstrated that a rotating cell-culture system (RCCS) can enhance the efficiency of human embryoid body (EB) formation and differentiation [13,14]. The rotating bioreactor of the RCCS creates a simulated microgravity, allowing the ES cells to grow and differentiate in three-dimensional (3D) culture [15]. Hepatocytes in 3D multicellular aggregates or spheroids have been shown to maintain their morphology and ultrastructural characteristics, resulting in higher degrees of liver-specific functions [16,17]. Previous reports have also revealed that mouse ES (mES) cells differentiate into hepatocytes via embryoid body (EB) formation *in vitro*[18,19]*.*

In our prior study, we demonstrated that the growth and differentiation of ES cells in a biodegradable polymer scaffold within a rotating microgravity bioreactor yields organized functional hepatocytes that can be used in research on bioartificial livers and engineered liver tissue [20]. Therefore, we hypothesized that a rotating bioreactor could be used for the efficient large-scale differentiation of ES cells into hepatocytes, which may further serve as an ideal source of cells for both hepatocyte transplantation and the creation of bioartificial livers. Here, to validate this hypothesis, we investigated whether mES cells can be induced to differentiate efficiently into cells with the morphologic, biologic, and functional characteristics of hepatocytes by using a rotating bioreactor and exogenous growth factors and hormones.

## Materials and methods

### ES cells culture

mES cells (CCE line) (Stemcell Technologies, Vancouver, BC, Canada) were cultured on gelatin-coated dishes in Iscove modified Dulbecco medium (IMDM) (Gibco, Grand Island, NY, USA) containing 15% fetal bovine serum (FBS) (Gibco), 0.1 m*M* nonessential amino acids, 2 m*M* L-glutamine, 0.1 m*M* monothioglycerol, 50 U/ml penicillin, 50 μg/ml streptomycin, and 1,000 U/ml recombinant mouse leukemia inhibitory factor (Chemicon International, Temecula, CA, USA).

### EB formation and hepatic differentiation in a rotating bioreactor

mES cells were dissociated by using trypsin and seeded into a rotating bioreactor (Synthecon Inc., Houston, TX, USA) at a concentration of 1 × 10^6^ cells/ml IMDM, which was supplemented with 10^-7^ *M* dexamethasone (Sigma, St. Louis, MO, USA), 1% dimethyl sulfoxide (DMSO) (Sigma), 20 ng/ml recombinant mouse hepatocyte growth factor (HGF) (R&D Systems, Minneapolis, MN, USA), 10 ng/ml recombinant human fibroblast growth factor-4 (FGF4) (Sigma), ITS (10 μg/ml insulin, 5 μg/ml transferrin, 5 ng/ml selenium) (Gibco), 15% FBS, 0.1 m*M* nonessential amino acids, 2 m*M* L-glutamine, 0.1 m*M* monothioglycerol, 50 U/ml penicillin, and 50 μg/ml streptomycin. The configuration of RCCS consists of four 50-ml-capacity bioreactors. The rotating bioreactor was set to rotate at a speed of 25 rpm at 37°C and with a humidified atmosphere containing 5% CO_2_, simulating microgravity to encourage cell 3D growth and differentiation. Every 3 to 4 days, 70% to 80% of the culture medium was replaced with fresh medium. In total, the culture duration was 21 days. mES cells were also differentiated by using the aforementioned exogenous growth factors and hormones in a culture plate, a two-dimensional culture (2D), as a control.

### Visualization of cell growth and tissue processing

At various time points, EBs were removed from the rotating bioreactor and placed into six-well plates, and observed by using a stereomicroscope (SZX12; Olympus, Tokyo, Japan). Subsequently, the EBs were cultured and grown in six-well plates in the same differentiation medium described earlier and observed by using a phase-contrast microscope (IX50; Olympus). For histologic examination, the EBs were fixed for 24 hours in 10% neutral buffered formalin, routinely processed, and embedded in paraffin. Serial sections (4 μm) were then cut and stained with hematoxylin and eosin (H&E).

### RNA extraction and RT-PCR

The total RNA was extracted by using an RNeasy Mini Kit (Qiagen, Valencia, CA, USA) and treated with RNase-free DNase (Qiagen). A reverse transcriptase-polymerase chain reaction (RT-PCR) was performed by using a Qiagen OneStep RT-PCR Kit with 10 U RNase inhibitor (Invitrogen, Carlsbad, CA, USA) and 20 ng of RNA, according to the manufacturer’s instructions. The PCR primers and the length of the amplified products were as follows: albumin (ALB) (5′-GCT ACG GCA CAG TGC TTG-3′, 5′-CAG GAT TGC AGA CAG ATA GT-3′; 55°C; 35 cycles; 260 bp); α-fetoprotein (AFP) (5′-TCG TAT TCC AAC AGG AGG-3′, 5′-AGG CTT TTG CTT CAC CAG-3′; 55°C; 35 cycles; 182 bp); tyrosine aminotransferase (TAT) (5′-ACC TTC AAT CCC ATC CGA-3′, 5′-TCC CGA CTG GAT AGG TAG-3′; 58°C; 35 cycles; 206 bp); glucose-6-phosphatase (G6P) (5′-CAG GAC TGG TTC ATC CTT-3′, 5′-GTT GCT GTA GTA GTC GGT-3′; 58°C; 35 cycles; 210 bp); transthyretin (TTR) (5′-CTC ACC ACA GAT GAG AAG-3′, 5′-GGC TGA GTC TCT CAA TTC-3′; 50°C; 35 cycles; 226 bp); and beta-actin (5′-TTC CTT CTT GGG TAT GGA AT-3′, 5′-GAG CAA TGA TCT TGA TCT TC-3′; 55°C; 35 cycles; 200 bp). The PCR products were separated by electrophoresis on 1.2% agarose gels and stained with ethidium bromide. Beta-actin was used as an endogenous control.

### Western blot (WB) analysis

The protein concentration of the EBs lysates was measured by using a Bio-Rad Protein Assay Kit (Bio-Rad, Hercules, CA, USA). Approximately 20 μg of protein from each supernatant was subjected to 4% to 15% Tris–HCl gel electrophoresis (Bio-Rad) and then transferred onto a nitrocellulose membrane (Pierce, Rockford, IL, USA). The membranes were blocked in 2.5% nonfat milk in phosphate-buffered saline (PBS), followed by incubation with a goat anti-mouse ALB antibody (1:10,000) (MP Biomedicals, Aurora, OH, USA), a monoclonal anti-human/mouse AFP antibody (1:1,000) (R&D Systems), or a monoclonal anti-mouse cytokeratin 18 (CK-18) antibody (1:2,500) (PROGEN Biotechnik, Heidelberg, Germany). The blots were then incubated with a horseradish peroxidase (HRP)-conjugated donkey anti-goat IgG or goat anti-mouse IgG secondary antibody (Santa Cruz Biotechnology, Santa Cruz, CA, USA). The protein bands were visualized by using enhanced chemiluminescence (Pierce), and images were captured by using medical x-ray film.

### Immunofluorescence

After deparaffinization and rehydration, the sections of the day-14 EBs were heated in a microwave at 100°C for 5 minutes in 10 m*M* sodium citrate for antigen retrieval, followed by blocking with 2% nonfat milk and 1% bovine serum albumin (BSA) in PBS for 45 minutes at room temperature. The slides were incubated with a goat anti-mouse ALB antibody (1:100) (Bethyl, Montgomery, TX, USA), a monoclonal anti-human/mouse AFP antibody (1:200) (R&D Systems), or a monoclonal against mouse CK18 antibody (1:500) (Progen Biotechnik) overnight at 4°C. The slides were then treated with biotinylated anti-mouse/rabbit IgG (Dako, Carpinteria, CA, USA) or biotinylated anti-goat IgG (Dako) for 60 minutes and followed by labeling with Texas Red-conjugated streptavidin (Jackson ImmunoResearch, West Grove, PA, USA) and DAPI (Sigma) nuclear staining. Immunofluorescence was examined with fluorescence microscopy.

For the immunocytochemistry analysis, day-14 EBs were plated on chamber slides and cultured for 5 days. Next, the EB-derived cells were fixed in 4% paraformaldehyde in PBS for 20 minutes at 4°C and permeabilized by using 0.1% Triton X-100 for 15 minutes. After blocking with a blocking solution, the cells were incubated with primary antibody, as described for the immunohistochemistry experiments, for 45 minutes at room temperature. The appropriate secondary antibody and Texas Red-conjugated streptavidin were then applied as described earlier.

### ALB product of cultured EBs

At days 7, 14, and 21 of the rotating culture, EBs were seeded in 12-well plates at approximately 50 EBs/well and incubated with fresh medium for 72 hours in 5% CO_2_ at 37°C. The amount of ALB secreted into the medium was quantified by using a mouse ALB ELISA quantitation kit (Bethyl) under the conditions recommended by the manufacturer.

### Cytochrome P450 activity assay

During the rotating culture, EBs were removed from the bioreactor at days 7, 14, and 21 and incubated with 10 μ*M* pentoxyresorufin (Sigma) for 60 minutes at 37°C. The red fluorescence from the resorufin in the EB-derived cells, which was produced from the O-dealkylation of pentoxyresorufin by cytochrome P450, was visualized by confocal laser scanning microscopy (CLSM) (IX 71, Olympus) [21]. The EBs were then immediately dissociated with trypsin, fixed in 4% paraformaldehyde in PBS, and analyzed by flow cytometry (BD Biosciences, Franklin Lakes, NJ, USA). Additionally, EBs were seeded and statically cultured in six-well plates for 4 days, and the resultant EB-derived cells were also incubated with pentoxyresorufin and examined by CLSM [22].

### Uptake of low-density lipoprotein (LDL)

EBs from day 7, 14, and 21 were incubated with 20 μg/ml DiI-Ac-LDL (Biomedical Technologies, Stoughton, MA, USA) for 4 hours at 37°C. The incorporation of the fluorochrome-labeled LDL into the EB-derived cells was visualized by CLSM. The flow-cytometric analysis [23] and CLSM observation of the DiI-Ac-LDL uptake by the EB-derived cells were performed in a similar manner to the assays described earlier for cytochrome P450 activity.

### Periodic acid-Schiff (PAS) staining

PAS staining for intracellular glycogen was performed for 72 hours on both EBs sections and the EBs cultured in six-well plates by using a PAS staining kit (American Master Tech Scientific, Lodi, CA, USA) [24].

### Indocyanine green (ICG) uptake

Rotating-culture EBs were removed from the bioreactor each week and further cultured in six-well plates for 72 hours. These EBs were then incubated in DMEM containing 0.1% ICG (Sigma) and 10% FBS for 30 minutes at 37°C. After rinsing 3 times with PBS, the cellular uptake of ICG was examined by both a stereomicroscopy and phase-contrast microscopy [25,26].

### Injection of EB-derived cells and culture supernatant

This study was approved by the Research Ethics Committee of the Southwest Hospital and Third Military Medical University. After 14 days of culture, the EBs were dissociated by using trypsin. These isolated cells were incubated with DiI-Cell-labeling Solution (Molecular Probes, Eugene, OR, USA) for 15 minutes at 37°C and then washed 5 times in PBS. The washed cells were resuspended in PBS at a concentration of 1 × 10^6^ cells/ml, and the resultant cell suspension (0.2 ml) was transplanted into the spleens of 6- to 8-week-old nude mice under anesthesia. The cell-culture supernatant from the same EBs samples (0.2 ml) was injected into the spleens of nude mice as a control. Each experimental group consisted of six animals. One and 2 months after the cell transplantation and culture-supernatant injection, the nude mice were killed, and their organs, including the liver, spleen, lung, and kidney, were removed. The presence of tumors in these organs was macroscopically investigated. Samples of tissues were fixed in formalin, stained with H&E, and then microscopically confirmed. Other tissue samples were placed into OCT compound, and frozen sections 10 μm) were prepared and observed by fluorescence microscopy.

### Statistical analysis

Statistical analyses were performed by using PRISM 5.01 (GraphPad Software). The data are presented as the mean values ± SD of *n* determinations, as indicated in the figure legends. ANOVA was used to calculate the significance of the differences between the mean values. A *P* value of < 0.05 was considered statistically significant.

## Results

### EB formation and hepatic differentiation

**Figure 1:**
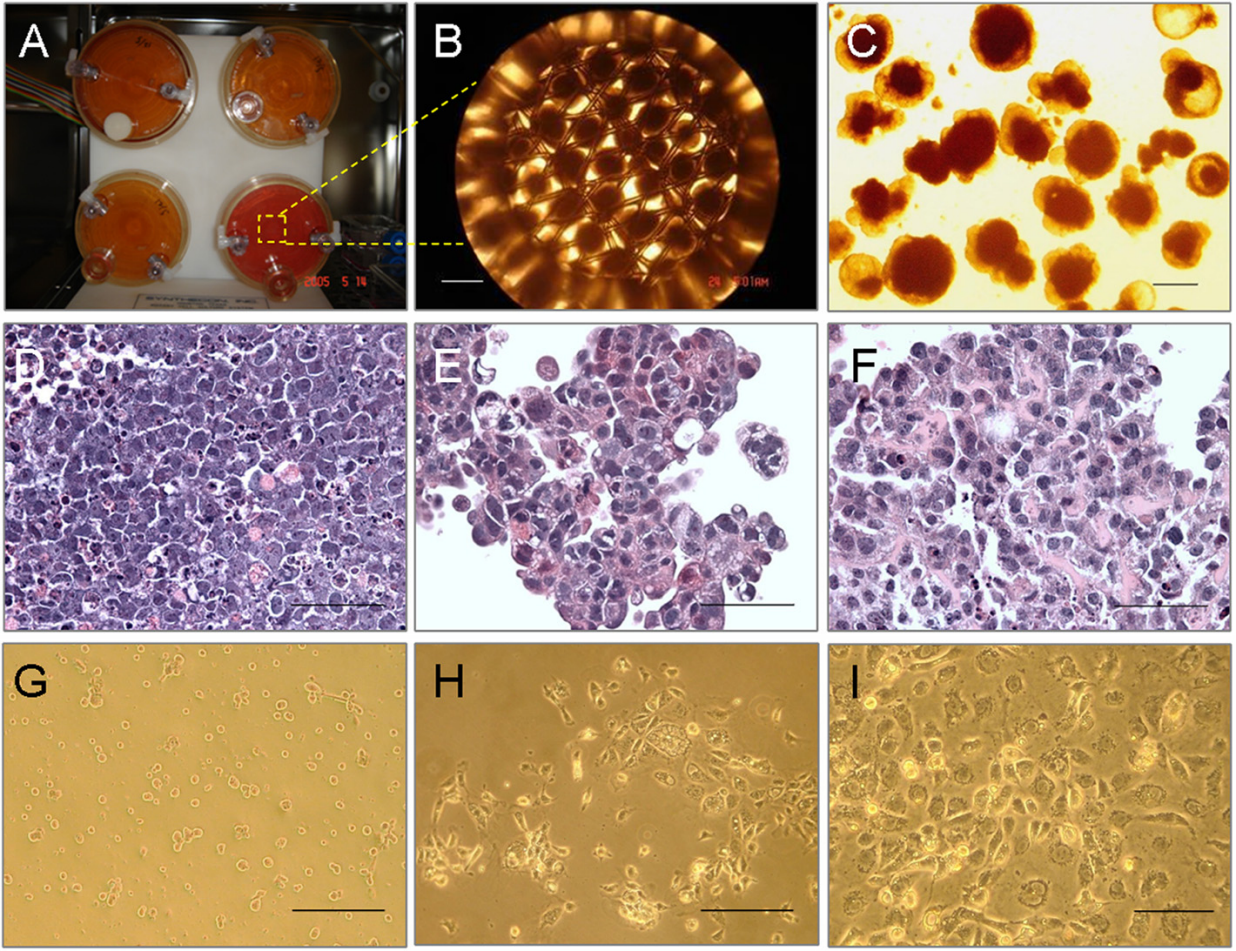
**Morphologic and histologic characteristics of EBs and EB-derived cells in the rotating bioreactor. (A)** A rotating bioreactor was used to create a 3D RCCS for mES cell culture. **(B)** A large number of homogeneous EBs was formed at day 7 in a rotating bioreactor with exogenous growth factors and hormones. **(C)** Image of EBs by stereomicroscopy at day 14. **(D-F)** Images of EBs with H&E staining at day 7 **(D)**, 14 **(E)**, 21 **(F)**. EB-derived cells from day 14 and day 21, but not day 7, exhibited polyhedral contours, large nuclei, and a cordlike arrangement. **(G-I)** Images of EB-derived cells. When the day 21 EB-derived cells were seeded in six-well plates **(G)**, most of the cells had a uniform pyramidal shape after 3 days **(H)** and 5 days **(I)** of culture. Scale bar, 100 μm.

To form EBs and differentiate these cells into hepatocytes, we used a rotating bioreactor (Figure 1A) to produce a 3D cell-culture system. mES cells were cultured in the simulated microgravity generated from the dynamic 3D system. When the mES cells were seeded at a concentration of 1 × 10^6^ cells/ml and rotated in the bioreactor at a speed of 25 rpm, cell aggregates were observed after 12 to 24 hours, and these aggregates became typical EBs within 1 week (Figure 1B). To investigate the cell number of EBs cultured in the bioreactor at day 21, EBs was dissociated by trypsin and counted by using a hemocytometer. The total number of cells harvested from every bioreactor was 3.2 ± 1.1 × 10^9^ at 21 days of culture.

Examination of the EBs under a stereomicroscopy revealed relatively homogeneous cell aggregates at day 7, with a few cystic EBs present (Figure 1C). Extensive cell clumps appeared after 2 to 3 weeks of suspension-culture. H&E staining clearly revealed the differentiation of the mES cells into cells with hepatocyte-like cells morphology. These cells exhibited polyhedral contours, large nuclei, and cordlike arrangement within the EBs at days 14 and 21 (Figure 1E, F), but not at day 7 (Figure 1D).

Further to confirm the hepatic differentiation of the ES cells in the EBs, we seeded the EBs in six-well plates and cultured the cells in the same differentiation medium (Figure 1G). Morphologically distinct cell types were observed in subculture. In particular, many of the day-21 EB-derived cells were characterized by a uniform pyramidal shape, large round nuclei, and dark granular deposits within the cytoplasm after 3 days (Figure 1H) and 5 days (Figure 1I) of culture, consistent with our histologic observations.

### Hepatic marker expression

**Figure 2:**
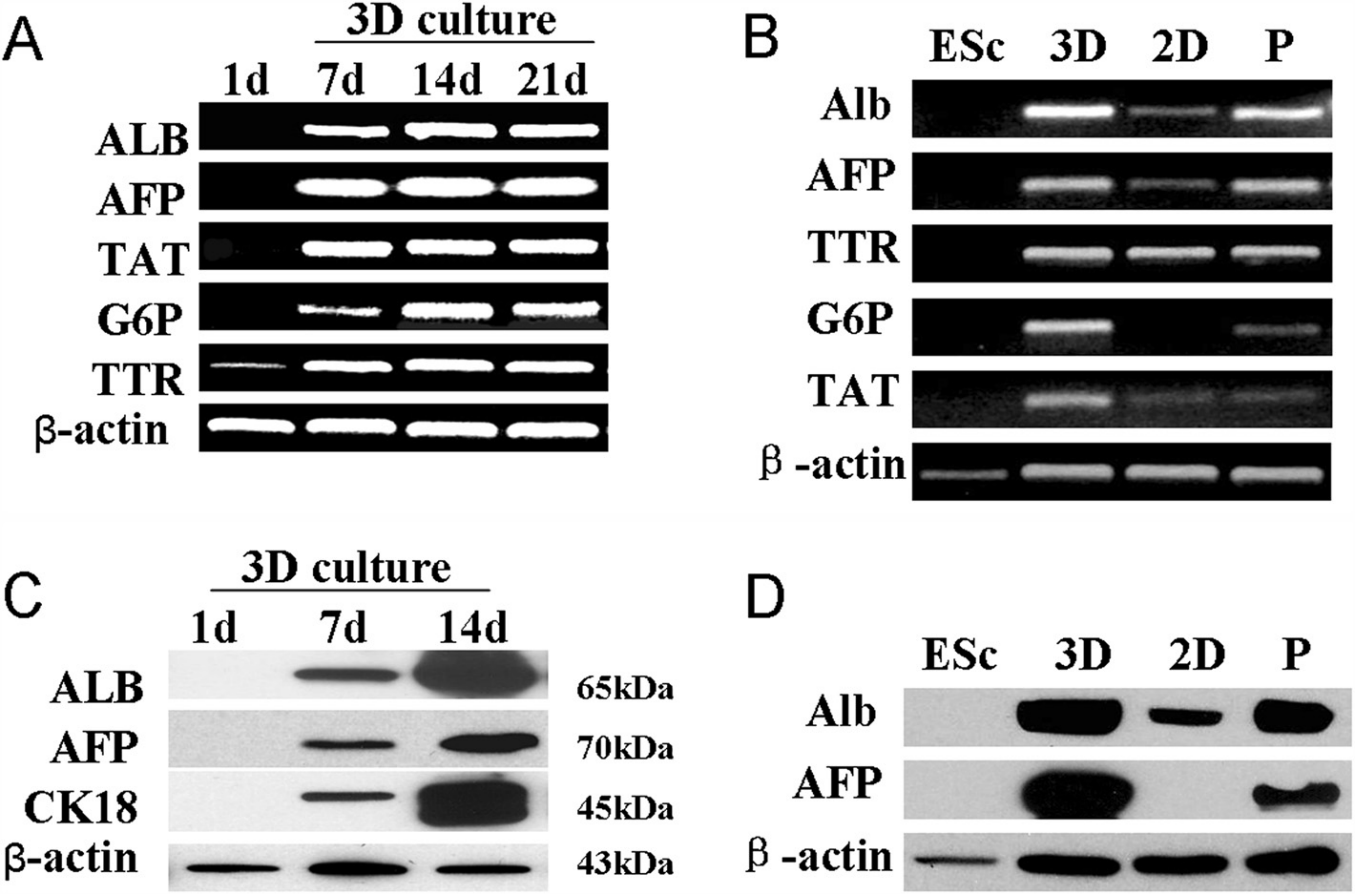
**Liver-specific gene expression of EBs.** The expression of the liver-specific genes in the EBs was analyzed by RT-PCR **(A, B)** and WB analysis **(C, D)**. **(A, C)** The expression of the hepatic genes **(A)** and proteins **(C)** in the EBs at different time point of culture. **(B, D)** The expression of the hepatic genes **(B)** and proteins **(D)** in the mES cells from the 3D culture and the differentiated mES cells from the 2D culture. P, positive control.

The ES cells were evaluated for the expression of hepatic-specific markers by using RT-PCR and WB analysis before and after differentiation. The steady-state levels of the ALB, AFP, TAT, G6P, and TTR mRNAs were determined in the mES cells and differentiating EBs every 7 days by RT-PCR. All of the mRNAs were detected in the EBs at day 7 and were maintained or increased up to day 21 of culture. Undifferentiated mES cells did not express ALB, AFP, TAT, or G6P mRNA, but TTR mRNA was detected (Figure 2A). In contrast to the differentiated mES cells grown in culture plates with the same exogenous growth factors and hormones, the EB cells cultured in the rotating bioreactor exhibited increased expression of hepatic-specific genes (Figure 2B). The day-1 EB-derived cells did not express ALB, AFP, or CK-18. However, protein bands corresponding to ALB, AFP, and CK-18 were visible in the EBs at day 7. The band intensity reached a maximum at day 14 (Figure 2C), and a significant difference in ALB and AFP expression was observed between the EB-derived cells cultured in the bioreactor and the mES cells differentiated in the 2D culture (Figure 2D).

### Immunohistochemical and immunocytochemical staining

**Figure 3:**
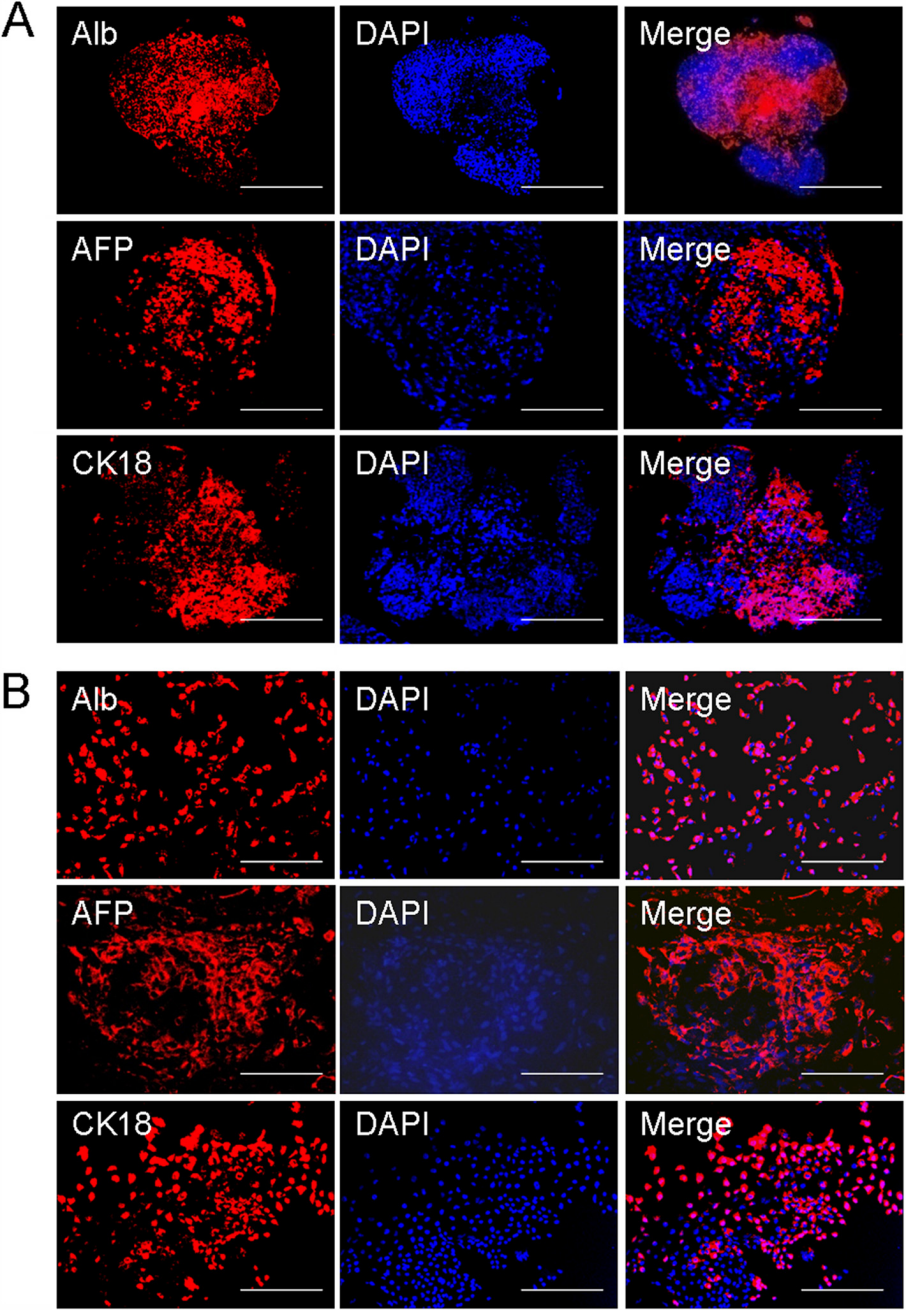
**Immunohistochemical and immunocytochemical staining of EBs from the rotating bioreactor. (A)** Immunohistochemical staining was performed on sections of day-14 EBs by using anti-ALB, anti-AFP, and anti-CK18 antibodies (red). DAPI was also included for nuclear staining (blue). **(B)** Immunocytochemical staining for ALB, AFP, and CK18 was performed on the day 14 EB-derived cells cultured on chamber slides for 5 days. Scale bar, 100 μm.

Further characterization of hepatic-specific protein expression was performed by using immunohistochemical and immunocytochemical staining. After 14 days of culture in the rotating bioreactor, ALB, AFP, and CK-18 were detected in paraffin sections of the EBs (Figure 3A). Immunocytochemical staining revealed that the day 14 EB-derived cells were positive for the three markers of mature hepatocytes after 3 days of monolayer subculture (Figure 3B), which is consistent with our immunohistochemical observations of the paraffin sections. These results suggest that the microgravity of rotating bioreactor, together with exposure to exogenous growth factors, strongly promotes the differentiation of ES cells into hepatocyte-like cells and the hepatic maturation of the differentiated cells.

### ALB production

**Figure 4:**
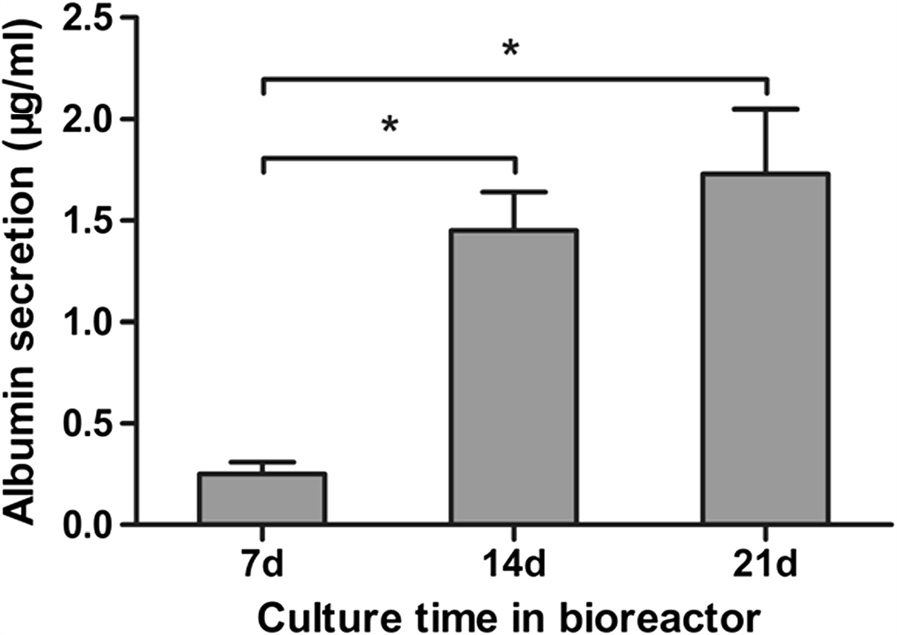
**ALB secretion from EB-derived cells.** ALB production by day 7, 14, and 21 EB-derived cells was quantified by using an ELISA kit after 72 hours of monolayer culture. The results are expressed as the means ± SD of six experiments. *P < 0.05.

ALB synthesis is a liver-specific parameter. Undifferentiated mES cells and 2D differentiated mES cells did not produce ALB at measurable levels (data not shown). Figure 4 showed that ALB was produced by EB-derived cells grown in a rotating bioreactor, after culture for 72 hours in 12-well plates. The day-14 and −21 EB-derived cells produced higher levels of ALB than the day-7 EB-derived cells (Figure 4).

### Metabolic enzyme activity

**Figure 5:**
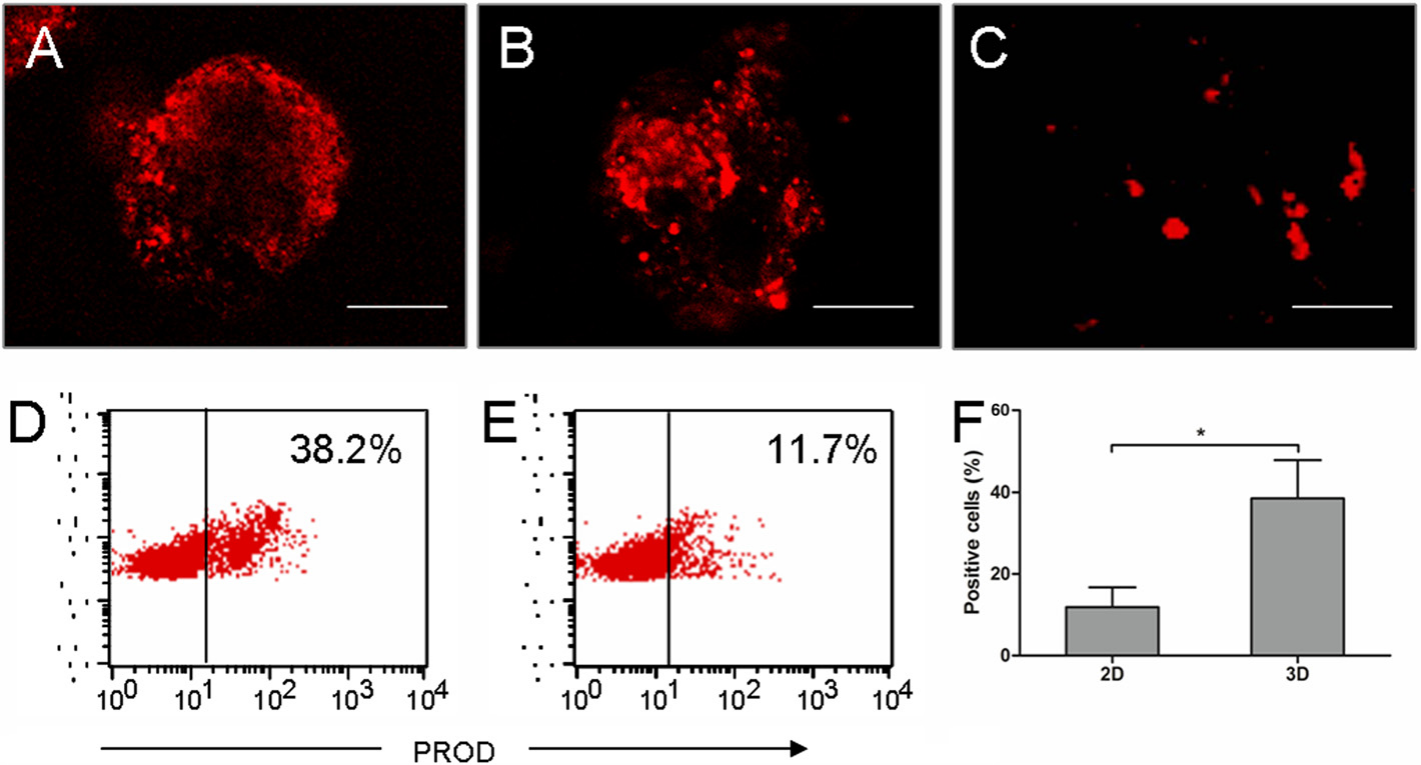
**Cytochrome P450 activity in EBs and EB-derived cells. (A-C)** PROD activity (red) was detected in day-21 EBs **(A)**, day-21 EB-derived cells grown in monolayer culture **(B)**, and mES cells differentiated in 2D culture **(C)** by CLSM. Scale bar, 100 μm. **(D-F)** Flow cytometric analysis showed that the number of PROD-positive day-21 EB-derived cells **(D)** was higher than the number of differentiated mES cells from the 2D culture **(E)**. A significant difference in the number of PROD-positive cells was noted between the differentiated cell samples from the 3D and 2D cultures **(F)**. *P < 0.05.

Xenobiotic metabolism, which is mediated mostly by cytochrome P450 enzymes, is a characteristic function of hepatocytes. Cytochrome P450 activity can be assessed by using the pentoxyresorufin-*O*-dealkylase (PROD) assay, in which pentoxyresorufin, a nonfluorescent compound, changed into resorufin, which emits red fluorescence. No PROD activity was observed in the aggregates of the undifferentiated mES cells or in the day-7 EBs. However, after 14 or 21 days in the rotating culture, the EBs exhibited marked PROD activity (Figure 5A). A significant level of fluorescence was also observed in the cells cultured from the day-14 and day-21 EBs (Figure 5B). In contrast, little fluorescence was noted in the mES cells that were grown in the six-well plates (Figure 5C). Flow-cytometric analysis confirmed that the rotating culture resulted in a significant increase in the number of PROD-positive cells in the EBs than among the mES cells differentiated in 2D culture (Figure 5D-F). These results suggest that because of the presence of endogenous P450 enzymes, the EB-derived cells were able to assume the metabolic functions of hepatocytes.

### LDL uptake

**Figure 6:**
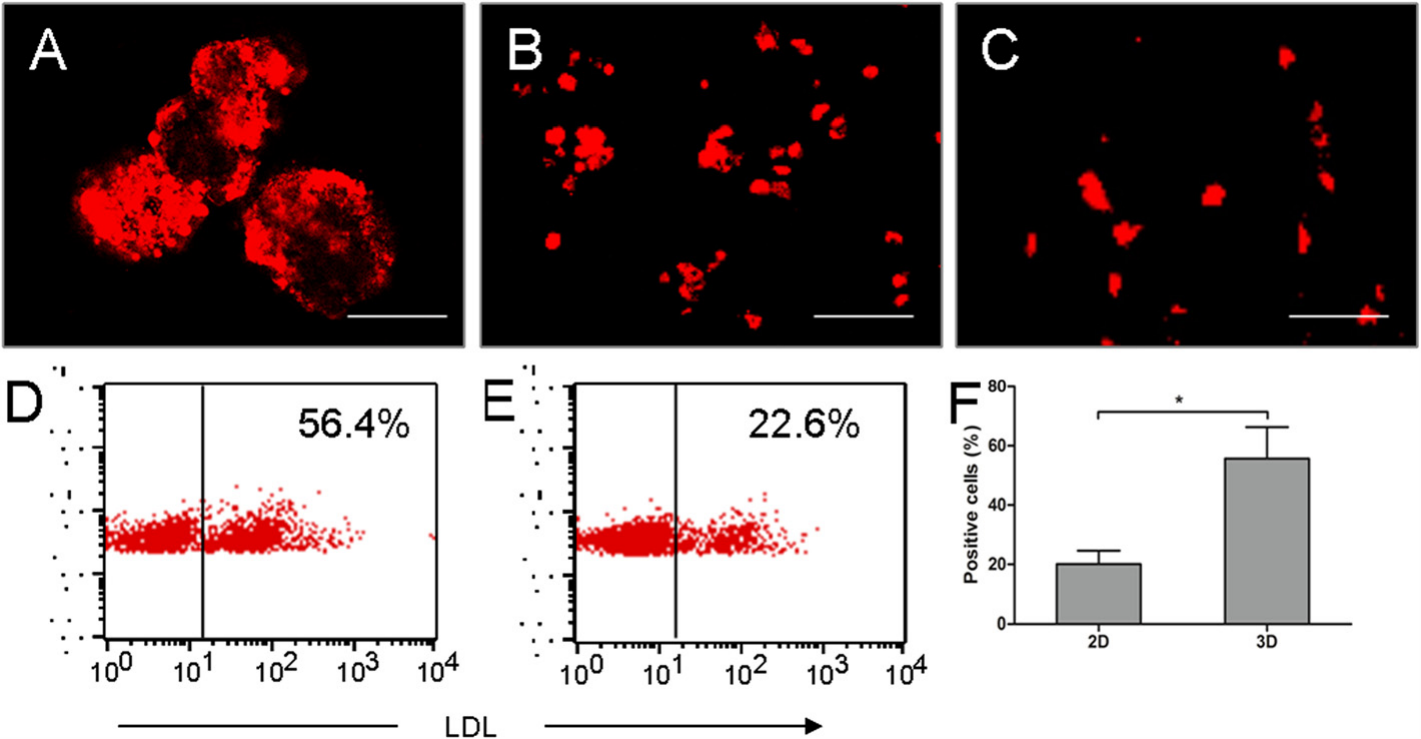
**LDL-uptake analysis of EBs and EB-derived cells. (A-C)** CLSM images indicated that the day-21 EBs **(A)** could incorporate DiI-Ac-LDL (red). Additionally, more day 21 EB-derived cells grown in monolayer culture incorporated DiI-Ac-LDL **(B)** than the mES cells differentiated in 2D culture **(C)**. Scale bar, 100 μm. **(D-F)** Flow-cytometric analysis showed that the number of LDL uptake-positive cells derived from day-21 EBs **(D)** was higher than the number of differentiated mES cells from 2D culture exhibiting LDL uptake **(E)**. A significant difference in the LDL uptake between the differentiated cell samples from the 3D and 2D cultures **(F)**. *P < 0.05.

The majority of LDL is metabolized in the liver. Thus, LDL uptake is an important function of hepatocytes. The ability of EB-derived cells to take up LDL was assessed by DiI-Ac-LDL staining and flow cytometry. Increased staining was observed in the day-14 and day-21 EBs (Figure 6A). Similar results were also observed for the staining of the statically cultured EB-derived cells (Figure 6B). The differentiated mES cells were able to incorporate LDL after 14 days of culture in plates, but these cells were low in quantity (Figure 6C). Flow-cytometric analysis showed that the number of LDL-positive EB-derived cells was greater than the number of LDL-positive mES cells from the 2D culture (Figure 6D-F).

### Glycogen and ICG staining

**Figure 7:**
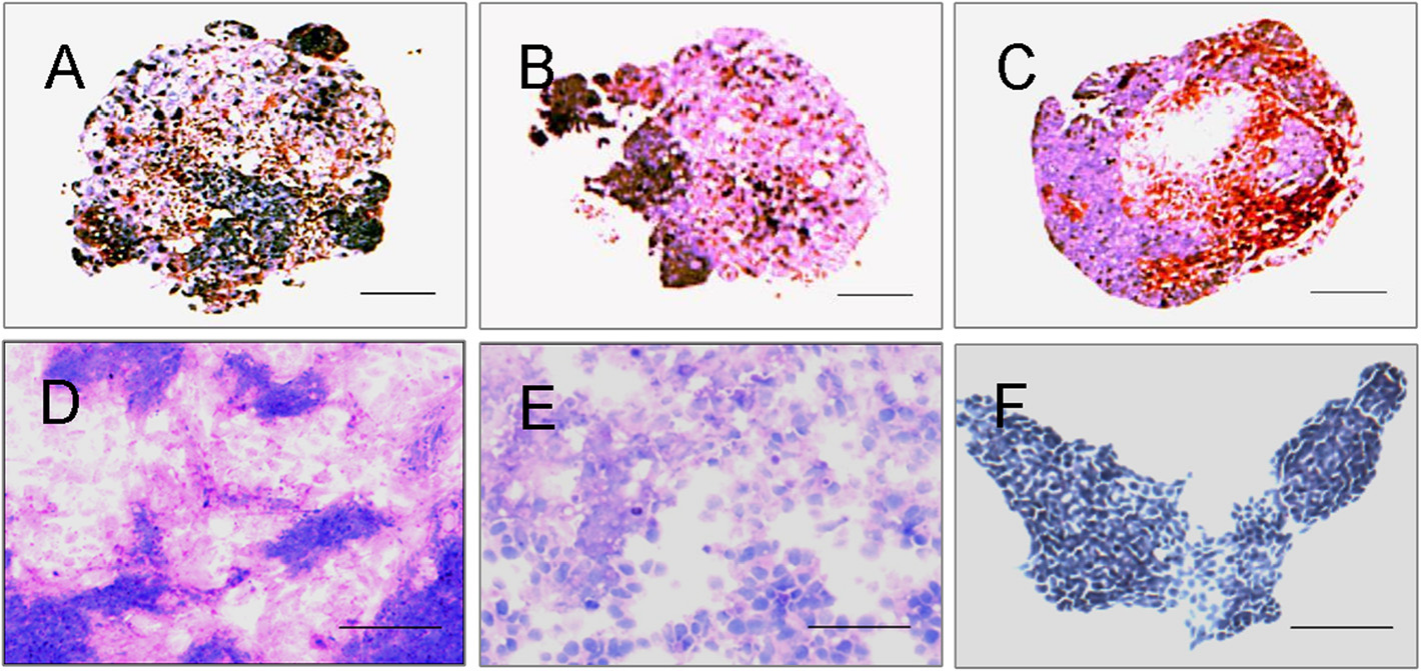
**PAS staining of EBs and EB-derived cells.** PAS staining, used to detect glycogen in the EB sections, showed that the number of positive cells increased from day 7 **(A)** to day 14 **(B)** and day 21 **(C)**. More PAS-positive cells were present among the day 21 EB-derived cells grown in monolayer culture **(D)** than the differentiated mES cells from the 2D culture **(E)**. In contrast, the undifferentiated mES cells were negative for PAS staining **(F)**. Scale bar, 50 μm.

**Figure 8:**
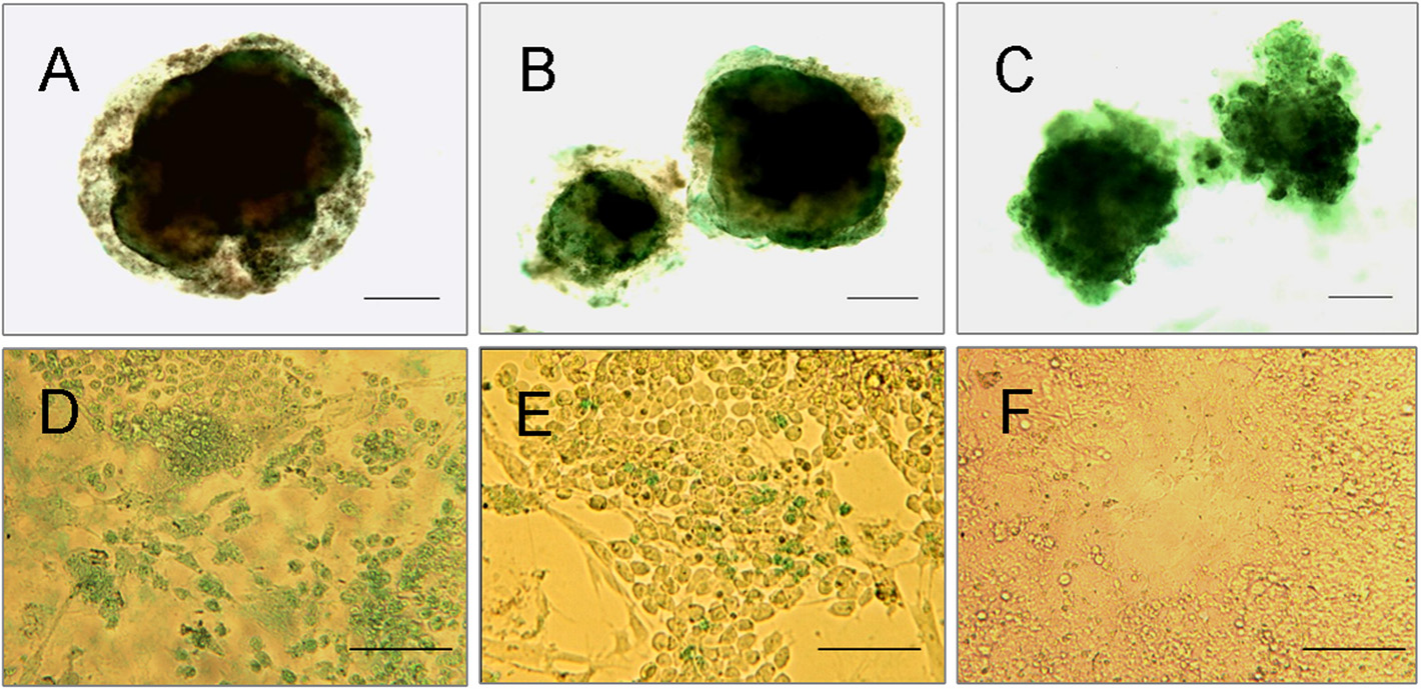
**Indocyanine green staining of EBs and EB-derived cells.** A low level of indocyanine green staining was found in the day 7 EB-derived cells **(A)**, whereas an increasing number of ICG-positive cells appeared at day 14 **(B)** and day 21 **(C)**. Additionally, more ICG-positive cells were present among the day 21 EB-derived cells grown in monolayer culture **(D)** compared with the differentiated mES cells from the 2D culture **(E)**. The undifferentiated mES cells were negative for ICG staining **(F)**. Scale bar, 50 μm.

Glycogen production, also known as gluconeogenesis, is another important function of hepatocytes. ICG is clinically used as a test substance to evaluate liver function, as this dye is eliminated exclusively by mature hepatocytes. To examine glycogen synthesis and storage in the differentiated EB-derived cells, PAS staining was performed on both EB sections and EBs that were cultured in six-well plates. Stored glycogen was not observed in the undifferentiated mES cells (Figure 7F) but was visualized in the EBs after 7 days of culture in the rotating bioreactor. The number of positive cells increased in a time-dependent manner (Figure 7A-C). These findings were confirmed by PAS staining of the EB-derived cells after 3 days of static subculture in plates (Figure 7D). Nevertheless, fewer PAS-positive cells were found in the differentiated mES cell population from the 2D culture than in the EB-derived cells after 3 days of static subculture in plates (Figure 7E). Similar to the results of the PAS staining, an increasing number of ICG-positive cells were detected in the EBs between day 7 and day 21 (Figure 8A-C). ICG uptake was also clearly apparent in the EB-derived cells after 3 days of static culture (Figure 8D). In the control experiment, only a few ICG-positive cells were observed among the differentiated mES cells from the 2D culture (Figure 8E), and the undifferentiated mES cells were negative for ICG staining (Figure 8F).

### Transplantation of EB-derived cells

**Figure 9:**
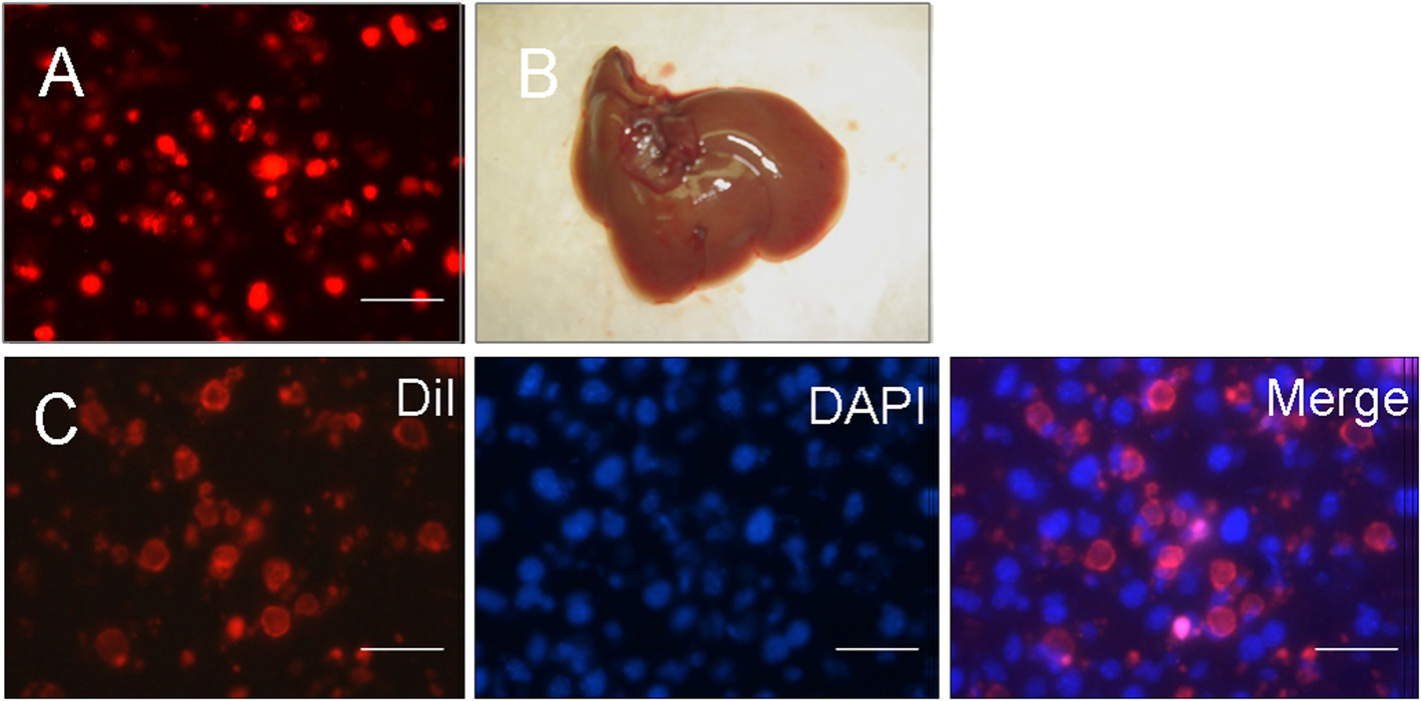
**Transplantation of EB-derived cells. (A)** Day 14 EB-derived cells labeled with DiI (red) were transplanted into the spleens of nude mice under anesthesia. **(B)** No development of tumors, such as teratomas, was observed in the livers of the recipient mice 2 months after the cell injection. **(C)** One month after transplantation, fluorescence from the EB-derived cells labeled with DiI (red) was detected in frozen sections of the recipient livers. Scale bar, 200 μm.

To assess whether the EB-derived cells were efficiently integrated into the livers of the recipient mice, we injected 14-day-old EB-derived cells labeled with DiI into the spleens of the nude mice (Figure 9A). Because the day-14 EBs not only strongly expressed hepatocyte-specific markers but also exhibited functions typical of hepatocytes, the cells isolated from these EBs were used for the injections. One month after transplantation, a large number of EB-derived cells labeled with DiI were detected in the recipient livers (Figure 9C), whereas no DiI-labeled EB-derived cells were detected in the recipient spleens. The results suggest that hepatocytes derived from EBs cultured in the rotating bioreactor could be engrafted into the livers of the recipient mice. One and two months after injection, no tumors, such as teratomas, were observed in the organs of the recipient mice (Figure 9B). We then injected the cell-culture supernatant from the day-14 EBs into nude mice but again did not find tumors in the organs of the mice 2 months after injection. Although these results indicate that hepatocytes or other cells originating from ES cells grown in a rotating bioreactor are safe and effective for use in our transplantation conditions, further studies using different animal models of liver diseases are needed to address the long-term safety, efficacy, and therapeutic potentials of hepatocytes derived from EBs in the rotating bioreactor.

## Discussion

Many hepatic differentiation protocols involve EB formation from ES cells grown in static culture [25,27]. Similar to embryos, EBs contain an endoderm, ectoderm, and mesoderm, which are important for the induction of complex differentiation [28]. Several techniques, such as the hanging-drop and conical-tube methods [29], have been developed to facilitate EB generation. The stirred-tank bioreactor and perfused 3D bioreactor have been applied for the hepatic differentiation of ES cells [30,31]. However, none of the current methods is sufficiently stable and controllable for the large-scale differentiation of ES cells for practical applications. Here, we used a rotating bioreactor to induce EB formation and hepatic differentiation. Cell culture in a rotating bioreactor has been shown to support the efficient, large-scale production of transplantable cells for bone-tissue engineering and of hematopoietic cells [32,33]. To the best of our knowledge, although a few studies have examined the formation and multiple-tissue or tissue-specific differentiation of EBs in a RCCS [15,34,35], no one has reported the hepatic differentiation of mES cells via EB formation in a rotating bioreactor. In our previous study [20], the hepatic differentiation of mES cells within the 3D condition of a rotating bioreactor has been described, which is similar to the present study. A 3D-culture system consisted of biodegradable scaffolds, growth-factor-reduced Matrigel, and a rotating bioreactor in our previous study. Scaffolds and Matrigel provided a 3D environment for the cell growth, proliferation, and differentiation of mES cells. Tissue mass with functional hepatocytes was obtained from the rotating bioreactor for bioartificial livers and engineered liver tissue. In this study, however, the formation of EBs was dependent on mES cell self-assembly. A native 3D microenvironment with cell-cell and cell-matrix interactions was recapitulated in EBs. A large number of EBs with functional hepatocytes were prepared in the rotating bioreactor. This method is suitable to large-scale generation of hepatocytes derived from ES cells for cell transplantation and the development of bioartificial livers. Therefore, our data indicate that the rotating bioreactor is an effective tool to provide a native 3D microenvironment for the large-scale production of hepatocytes derived from ES cells.

Several studies demonstrated that the optimal speed for EB formation is 15 to 20 rpm in the rotating bioreactor [33,36]. To enhance the efficiency of EB formation, we chose a slow rotational speed and a high cell concentration, resulting in large numbers of homogeneous EBs within 1 week. By culturing ES cells in suspension in this dynamic and mild environment, which is characterized by extremely low fluid shear stress and optimal nutrient and gas exchange, our protocol for EB formation not only avoids massive cellular damage but also controls the extent of EB agglomeration, thus reducing large central necrosis in EBs. More important, both histologic and morphologic observations revealed that this setup is more beneficial for the differentiation of ES cells into hepatocytes than standard conditions.

It has been previously shown that exogenous growth factors and hormones play an important role in the differentiation of ES cells into hepatocytes and in the expression of the hepatocyte phenotype [37]. However, it is still unclear which growth factor, hormone, or combination of the two is critical for the hepatic differentiation of ES cells. Additionally, the optimal timing for the use of these factors and hormones to differentiate ES cells into hepatocytes is not known. In the current study, we found that the hepatic differentiation of ES cells in a rotating bioreactor was more efficient with than without the presence of HGF, FGF4, dexamethasone, DMSO, and ITS. More specifically, the early use of medium containing these factors resulted in the efficient differentiation of ES cells into hepatocytes, as determined by morphologic analysis (data not shown).

In this study, we investigated the expression of TTR, AFP, ALB, G6P, and TAT at the mRNA level. Among these genes, TTR is a marker of endodermal differentiation, AFP represents endodermal differentiation as well as the early differentiation of fetal hepatocytes [38], ALB appears in early fetal hepatocytes and reaches a maximal level in adult hepatocytes [38], G6P is predominantly expressed in hepatocytes in the late gestational and perinatal stages [39], and TAT is an excellent enzymatic marker for perinatal or postnatal hepatocyte-specific differentiation and represents an important function of the liver [40]. In general, the expression of these specific genes in cells derived from EBs is characteristic of differentiated hepatocyte-like cells, although a proportion of these genes is also expressed in the yolk sac and other cells. The expression of such genes in EBs differentiating in the presence of growth factors parallels the expression observed in the embryonic liver *in vivo*. However, in our tests, the mRNAs of TTR, AFP, ALB, G6P, and TAT were expressed by day 7, and the expression of most of the genes, such as ALB, G6P, and TAT, was noticeable earlier than in previous reports [27,41]. The level expression of these genes was higher in the EB-derived cells cultured in the bioreactor than the mES cells differentiated in the 2D culture. Because the expression of the ALB, AFP, and CK18 proteins was also detected in the day-7 EBs by WB analysis, and based on our immunostaining results, we believe that growth of ES cells in a rotating bioreactor supplemented with exogenous growth factors not only strongly promotes differentiation into hepatocyte-like cells but also enhances cell maturation.

The assay for ALB synthesis is well known as a relatively specific test for the presence and activity of hepatocytes during the hepatic differentiation of stem cells [23,25]. We examined the ALB synthesis of mES cell-derived EBs to confirm the differentiation of the ES cells into functional hepatocytes. However, we failed to detect ALB directly at measurable levels during the rotating bioreactor culture period. As the probable reason for this issue was the relatively low cell concentration in the bioreactor, we removed the EBs from the bioreactor and seeded them in 12-well plates at a concentration of approximately 50 EBs/well. After incubation with fresh medium for 72 hours, these EBs secreted ALB into the culture medium. The ability of the EBs to synthesize ALB at days 14 and 21 was approximately sixfold to eightfold higher than at day 7, confirming the hepatic differentiation of the EBs.

Because normal hepatocytes possess many complex functions, in addition to our ALB analysis, we used other independent functional assays to verify further the hepatocyte-like attributes of the differentiated ES cells. Four functional tests were performed on both the EBs from the rotating culture and the EB-derived cells from the static culture more precisely to characterize the hepatic functions of the differentiated cells. Our data demonstrate that the EB-derived cells possessed cytochrome P450 activity, stored glycogen, and took up ICG and LDL, which were higher than that of cells derived from 2D culture. Together, all of these results strongly suggest that the cells differentiated in the rotating bioreactor, along with growth factors and hormones, were functional hepatocytes.

## Conclusion

The use of a rotating bioreactor for the differentiation of functional hepatocytes from ES cells, as reported in this study, has two advantages. First, the hepatic differentiation of ES cells in a rotating bioreactor is more effective than in traditional 2D culture. Second, large quantities of differentiated cells can be generated by using this technique. In the present study, most of the differentiated cells exhibited the complex functions of mature hepatocytes and the 3D characteristics of multicellular aggregates, conforming to the requirements of high-quality, high-density hepatocyte cultures for both hepatocyte transplantation and the seeding of bioartificial liver devices. Further research is needed to investigate the therapeutic potential of the differentiated cells grown under these conditions and to optimize the protocol for the differentiation of the EBs to avoid necrosis.

## Abbreviations

AFP: α-fetoprotein; ALB: Albumin; ALF: Acute liver failure; BSA: Bovine serum albumin; CK-18: Cytokeratin 18; CLSM: Confocal laser scanning microscopy; DMSO: Dimethyl sulfoxide; EB: Embryoid body; EBs: Embryoid bodies; ES: Embryonic stem; FBS: Fetal bovine serum; FGF4: Fibroblast growth factor-4; G6P: Glucose-6-phosphatase; H&E: Hematoxylin and eosin; HGF: Hepatocyte growth factor; HRP: Horseradish peroxidase; ICG: Indocyanine green; IMDM: Iscove modified Dulbecco medium; ITS: Insulin-transferrin-selenium; LDL: Low-density lipoprotein; mES: mouse ES; PAS: Periodic acid-Schiff; PBS: Phosphate-buffered saline; PROD: Pentoxyresorufin-*O*-dealkylase; RCCS: Rotating cell-culture system; TAT: Tyrosine aminotransferase; TTR: Transthyretin; WB: Western blot.

## Competing interests

The authors declare that they have no competing interests.

## Authors’ contributions

YW and YG conceived the idea and designed the experiments, SZ and YZ executed all the experiments, and LC and TL interpreted and analyzed the data and drafted the manuscript. YL was responsible for collection and assembly of data. All authors read and approved the final manuscript.
